# Supplementary material for: Investigating the Life Expectancy at Birth of Companion Dogs in Portugal Using Official National Registry Data
Source: Animals (Basel). 2024 Jul 23;14(15):2141. doi: 10.3390/ani14152141 (PMC11311093; doi:10.3390/ani14152141)
Supplement: Supplementary file 1 [file animals-14-02141-s001.zip › animals-3085467-supplementary.pdf]

Table S1: Current life table of female Portuguese companion dogs registered in SIAC (all pure breeds and crossbreed combined).

Age interval in years (x. x + 1)	Number living at age x (lx)	Number dying in (x.x+1) (dx)	Conditional Probability of death (qx)	Proportion surviving to age x	Number of dog-years lived at age x L(x)	Total number of dog-years lived from year x T(x)	Life expectancy for dogs in the age interval (x. x + 1) e(x)
0 - 1	138325	1751	0.01	1.00	99371	894574	8.95 (8.51 - 9.39)
1 – 2	136574	4275	0.03	0.99	97220	795203	8.05 (7.40 - 8.71)
2 – 3	132299	6892	0.05	0.96	93269	697982	7.29 (6.50 - 8.09)
3 - 4	125407	8587	0.07	0.91	87833	604713	6.66 (5.79 - 7.52)
4 – 5	116820	9394	0.08	0.85	81547	516880	6.09 (5.20 - 6.98)
5 – 6	107426	9802	0.09	0.78	74853	435333	5.56 (4.66 - 6.46)
6 – 7	97624	10109	0.10	0.71	67922	360480	5.05 (4.14 - 5.95)
7 – 8	87515	10411	0.11	0.64	60789	292558	4.54 (3.63 - 5.45)
8 – 9	77104	11034	0.13	0.57	53356	231769	4.05 (3.13 - 4.98)
9 - 10	66070	11665	0.16	0.50	45519	178414	3.60 (2.64 - 4.56)
10 – 11	54405	11783	0.20	0.42	37446	132894	3.20 (2.22 - 4.19)
11 – 12	42622	11384	0.24	0.33	29457	95448	2.86 (1.84 - 3.87)
12 – 13	31238	9990	0.28	0.26	22005	65991	2.59 (1.54 - 3.63)
13 - 14	21248	7827	0.31	0.18	15611	43986	2.38 (1.32 - 3.44)
14 – 15	13421	5390	0.33	0.13	10606	28375	2.23 (1.17 - 3.29)
15 – 16	8030	3563	0.36	0.08	6937	17769	2.10 (1.03 - 3.17)
16 – 17	4467	2061	0.37	0.05	4386	10833	2.01 (0.95 - 3.06)
17 – 18	2407	1133	0.38	0.03	2732	6446	1.91 (0.88 - 2.94)
18 - 19	1273	636	0.40	0.02	1671	3715	1.78 (0.78 - 2.78)
19 – 20	638	317	0.40	0.01	1005	2044	1.63 (0.71 - 2.55)
20 – 21	321	196	0.47	0.01	579	1039	1.38 (0.49 - 2.26)
21 - 22	125	100	0.57	0.00	287	460	1.15 (0.26 - 2.03)
22 - 23	25	25	1.00	0.00	173	173	1.00 (0.01 - 1.99)

Table S2: Current life table of male Portuguese companion dogs registered in SIAC (all pure breeds and crossbreed combined).

Age interval in years (x. x + 1)	Number living at age x (lx)	Number dying in (x.x+1) (dx)	Conditional Probability of death (qx)	Proportion surviving to age x	Number of dog-years lived at age x L(x)	Total number of dog-years lived from year x T(x)	Life expectancy for dogs in the age interval (x. x + 1) e(x)
0 - 1	154824	5256	0.03	1.00	98331	849685	8.50 (7.80 - 9.20)
1 – 2	149569	8613	0.06	0.97	93957	751778	7.78 (6.91 - 8.65)
2 – 3	140956	9821	0.07	0.91	88180	657821	7.21 (6.29 - 8.12)
3 - 4	131134	10091	0.07	0.85	81954	569642	6.69 (5.77 - 7.61)
4 – 5	121044	10174	0.08	0.79	75623	487687	6.19 (5.27 - 7.11)
5 – 6	110870	9949	0.09	0.72	69334	412064	5.69 (4.78 - 6.59)
6 – 7	100921	10203	0.10	0.66	63037	342730	5.18 (4.27 - 6.08)
7 – 8	90718	10320	0.11	0.60	56629	279693	4.67 (3.77 - 5.58)
8 – 9	80398	10959	0.13	0.53	50001	223063	4.18 (3.25 - 5.10)
9 - 10	69439	11351	0.15	0.47	43072	173063	3.71 (2.78 - 4.65)
10 – 11	58088	11960	0.19	0.40	35860	129990	3.29 (2.31 - 4.26)
11 – 12	46127	11618	0.22	0.32	28570	94130	2.93 (1.93 - 3.93)
12 – 13	34510	10512	0.26	0.25	21672	65560	2.63 (1.60 - 3.65)
13 - 14	23998	8670	0.31	0.18	15560	43889	2.39 (1.34- 3.44)
14 – 15	15327	6285	0.34	0.13	10580	28329	2.22 (1.15 - 3.29)
15 – 16	9042	3989	0.36	0.08	6891	17749	2.11 (1.04 - 3.18)
16 – 17	5053	2350	0.38	0.05	4357	10858	2.02 (0.95- 3.09)
17 – 18	2703	1260	0.38	0.03	2712	6500	1.94 (0.91 - 2.98)
18 - 19	1443	636	0.36	0.02	1704	3789	1.82 (0.88 - 2.77)
19 – 20	807	417	0.41	0.01	1056	2084	1.57 (0.66 - 2.48)
20 – 21	390	232	0.46	0.01	604	1028	1.31 (0.49 -2.14)
21 - 22	158	158	1.00	0.00	424	424	1.00 (0.02-1.98)

Table S3: Current life table of crossbreed dogs registered in SIAC.

Age interval in years (x. x + 1)	Number living at age x (lx)	Number dying in (x.x+1) (dx)	Conditional Probability of death (qx)	Proportion surviving to age x	Number of dog-years lived at age x L(x)	Total number of dog-years lived from year x T(x)	Life expectancy for dogs in the age interval (x. x + 1) e(x)
0 - 1	83970	655	0,01	1.00	99612	948462	9.48 (9.13 – 9.84)
1 – 2	83315	1174	0,01	0.99	98529	848850	8.55 (8.10 – 9.01)
2 – 3	82141	3032	0,04	0.98	96062	750321	7.67 (6.98 – 8.36)
3 - 4	79109	5516	0,07	0.94	91112	654259	6.94 (6.04 – 7.83)
4 – 5	73593	6026	0,08	0.88	84477	856706	6.40 (5.48 – 7.33)
5 – 6	67567	5900	0,08	0.81	77630	478670	5.91 (5.00 – 6.82)
6 – 7	61667	5899	0,09	0.74	70852	401040	5.40 (4.50 – 6.31)
7 – 8	55768	5979	0,10	0.67	64031	892326	4.89 (3.99 – 5.80)
8 – 9	49789	6348	0,12	0.61	56967	266158	4.39 (3.47 – 5.31)
9 - 10	43441	6582	0,14	0.53	49579	911567	3.92 (2.99 – 4.86)
10 – 11	36859	6941	0,17	0.46	41880	159612	3.48 (2.51 – 4.45)
11 – 12	29918	6946	0,21	0.38	33991	927650	3.10 (2.10 – 4.10)
12 – 13	22972	6235	0,24	0.30	26455	83741	2.79 (1.78 – 3.79)
13 - 14	16737	5456	0,28	0.23	19661	57286	2.51 (1.48 - 3.53)
14 – 15	11281	4171	0,31	0.16	13889	37625	2.29 (1.26 – 3.32)
15 – 16	7110	3077	0,36	0.11	9307	23737	2.10 (1.04 – 3.15)
16 – 17	4033	1916	0,38	0.07	5894	14429	1.98 (0.91 – 3.04)
17 – 18	2117	1051	0,40	0.04	3600	8536	1.90 (0.84 – 2.96)
18 - 19	1066	511	0,39	0.03	2184	4935	1.82 (0.83 – 2.82)
19 – 20	555	289	0,41	0.02	1318	2752	1.66 (0.69 – 2.62)
20 – 21	266	149	0,44	0.01	762	1434	1.47 (0.57 -2.37)
21 - 22	117	82	0,52	0.01	407	672	1.22 (0.37 -2.08)
22 - 23	35	35	1,00	0.00	265	265	1.00 (0.02 -1.98)

Table S4: Current life table of Portuguese Podengos registered in SIAC.

Age interval in years (x. x + 1)	Number living at age x (lx)	Number dying in (x.x+1) (dx)	Conditional Probability of death (qx)	Proportion surviving to age x	Number of dog-years lived at age x L(x)	Total number of dog-years lived from year x T(x)	Life expectancy for dogs in the age interval (x. x + 1) e(x)
0 - 1	77683	752	0.01	1.00	99518	796052	7.96 (7.60 - 8.32)
1 – 2	76931	3922	0.05	0.99	96575	696534	7.03 (6.26 - 7.80)
2 – 3	73009	6191	0.08	0.94	90285	599959	6.37 (5.43 - 7.31)
3 - 4	66818	6542	0.09	0.86	82422	509674	5.90 (4.93 - 6.86)
4 – 5	60276	6603	0.10	0.78	74317	427251	5.45 (4.48 - 6.43)
5 – 6	53673	6588	0.12	0.70	66184	352935	5.02 (4.04 - 6.01)
6 – 7	47085	6347	0.13	0.62	58200	286750	4.62 (3.64 - 5.59)
7 – 8	40738	6216	0.14	0.54	50430	228550	4.21 (3.23 - 5.19)
8 – 9	34522	6124	0.16	0.47	42787	178120	3.82 (2.82 - 4.82)
9 - 10	28398	6007	0.19	0.39	35263	135333	3.47 (2.44 - 4.50)
10 – 11	22391	5539	0.22	0.32	28062	100070	3.17 (2.12 - 4.22)
11 – 12	16852	4769	0.25	0.25	21543	72008	2.93 (1.86 - 4.00)
12 – 13	12083	3912	0.28	0.18	15918	50465	2.73 (1.64 - 3.82)
13 - 14	8171	2948	0.31	0.13	11302	34547	2.59 (1.47 - 3.71)
14 – 15	5223	1915	0.31	0.09	7828	23245	2.51 (1.41 - 3.61)
15 – 16	3308	1174	0.30	0.06	5430	15417	2.41 (1.36 - 3.46)
16 – 17	2134	814	0.32	0.04	3751	9987	2.24 (1.21 - 3.27)
17 – 18	1320	504	0.32	0.03	2549	6236	2.05 (1.09 - 3.02)
18 - 19	816	367	0.37	0.02	1684	3687	1.79 (0.85 - 2.73)
19 – 20	449	232	0.41	0.01	1037	2004	1.54 (0.65 - 2.42)
20 – 21	217	143	0.50	0.01	579	966	1.26 (0.41 - 2.10)
21 - 22	74	74	1.00	0.00	388	388	1.00 (0.02 - 1.98)

Table S5: Current life table of Labradors Retriever registered in SIAC.

Age interval in years (x. x + 1)	Number living at age x (lx)	Number dying in (x.x+1) (dx)	Conditional Probability of death (qx)	Proportion surviving to age x	Number of dog-years lived at age x L(x)	Total number of dog-years lived from year x T(x)	Life expectancy for dogs in the age interval (x. x + 1) e(x)
0 - 1	13263	427	0.03	1.00	98415	976523	9.77 (9.03 - 10.50)
1 – 2	12836	496	0.04	0.97	94994	878108	9.07 (8.29 - 9.84)
2 – 3	12339	412	0.03	0.93	91626	783115	8.41 (7.72 - 9.10)
3 - 4	11927	451	0.04	0.90	88424	691489	7.68 (6.98 - 8.37)
4 – 5	11476	483	0.04	0.87	84964	603065	6.95 (6.26 - 7.65)
5 – 6	10993	530	0.05	0.83	81216	518101	6.23 (5.53 - 6.93)
6 – 7	10463	593	0.06	0.79	77073	436885	5.51 (4.81 - 6.22)
7 – 8	9870	712	0.07	0.75	72282	359812	4.80 (4.07 - 5.53)
8 – 9	9158	882	0.09	0.70	66477	287529	4.13 (3.36 - 4.89)
9 - 10	8276	1079	0.12	0.63	59404	221053	3.49 (2.69 - 4.30)
10 – 11	7197	1316	0.17	0.56	50878	161649	2.91 (2.07 - 3.75)
11 – 12	5881	1538	0.23	0.46	40880	110771	2.40 (1.52 - 3.28)
12 – 13	4343	1627	0.32	0.36	29928	69891	1.97 (1.05 - 2.88)
13 - 14	2716	1365	0.40	0.24	19438	39963	1.64 (0.71 - 2.58)
14 – 15	1351	862	0.48	0.15	11032	20525	1.41 (0.47 - 2.35)
15 – 16	489	373	0.55	0.08	5439	9493	1.26 (0.30 - 2.23)
16 – 17	116	94	0.58	0.03	2393	4054	1.20 (0.24 - 2.17)
17 – 18	22	17	0.57	0.01	1017	1661	1.17 (0.26 - 2.07)
18 - 19	5	4	0.63	0.01	419	644	1.05 (0.14 - 1.96)
19 – 20	1	1	1.00	0.00	225	225	1.00 (0.12 - 1.88)

Table S6: Current life table of German Shepherds registered in SIAC.

**Age interval in years (x. x + 1)**	**Number living at age x (lx)**	**Number dying in (x.x+1) (dx)**	**Conditional Probability of death (qx)**	**Proportion surviving to age x**	**Number of dog-years lived at age x L(x)**	**Total number of dog-years lived from year x T(x)**	**Life expectancy for dogs in the age interval (x. x + 1) e(x)**
0 - 1	11542	481	0.04	1.00	97960	831139	8.31 (7.55 - 9.08)
1 – 2	11061	563	0.05	0.96	93537	733180	7.64 (6.84 - 8.45)
2 – 3	10498	569	0.05	0.91	88750	639643	7.02 (6.22 - 7.81)
3 - 4	9929	592	0.06	0.86	83846	550893	6.38 (5.59 - 7.17)
4 – 5	9337	664	0.07	0.81	78552	467047	5.74 (4.93 - 6.55)
5 – 6	8673	666	0.07	0.76	72956	388495	5.13 (4.34 - 5.91)
6 – 7	8007	732	0.09	0.70	67089	315539	4.50 (3.71 - 5.29)
7 – 8	7275	796	0.10	0.64	60701	248451	3.88 (3.09 - 4.67)
8 – 9	6479	997	0.14	0.57	53282	187749	3.27 (2.44 - 4.11)
9 - 10	5482	1179	0.19	0.49	44407	134468	2.73 (1.86 - 3.61)
10 – 11	4303	1327	0.27	0.40	34336	90060	2.27 (1.35 - 3.20)
11 – 12	2976	1180	0.33	0.29	24237	55724	1.92 (0.99 - 2.85)
12 – 13	1796	958	0.42	0.19	15340	31488	1.62 (0.66 - 2.58)
13 - 14	838	552	0.50	0.11	8462	16147	1.44 (0.45 - 2.42)
14 – 15	286	207	0.53	0.06	4166	7686	1.35 (0.36 - 2.35)
15 – 16	79	57	0.53	0.03	1953	3519	1.32 (0.36 - 2.29)
16 – 17	22	16	0.53	0.01	916	1566	1.25 (0.34 - 2.17)
17 – 18	6	5	0.59	0.01	411	650	1.12 (0.22 - 2.01)
18 - 19	1	1	1.00	0.00	239	239	1.00 (0.02 - 1.98)

Table S7: Current life table of Brittany Spaniel dogs registered in SIAC.

Age interval in years (x. x + 1)	Number living at age x (lx)	Number dying in (x.x+1) (dx)	Conditional Probability of death (qx)	Proportion surviving to age x	Number of dog-years lived at age x L(x)	Total number of dog-years lived from year x T(x)	Life expectancy for dogs in the age interval (x. x + 1) e(x)
0 - 1	7903	18	0.00	1.00	99888	1024730	10.25 (10.05 - 10.45)
1 – 2	7885	51	0.01	1.00	99454	924842	9.27 (8.95 - 9.59)
2 – 3	7834	110	0.01	0.99	98441	827013	8.34 (7.90 - 8.79)
3 - 4	7724	227	0.03	0.98	96332	729528	7.46 (6.86 - 8.07)
4 – 5	7497	427	0.06	0.95	92286	633682	6.68 (5.89 - 7.47)
5 – 6	7070	547	0.07	0.90	86320	541696	6.04 (5.17 - 6.91)
6 – 7	6523	603	0.09	0.83	79318	455376	5.49 (4.59 - 6.39)
7 – 8	5920	587	0.09	0.76	72079	376059	4.97 (4.10 - 5.85)
8 – 9	5333	659	0.12	0.69	64520	303979	4.44 (3.52 - 5.35)
9 - 10	4674	641	0.13	0.61	56650	239459	3.96 (3.06 - 4.85)
10 – 11	4033	745	0.17	0.53	48305	182809	3.46 (2.51 - 4.42)
11 – 12	3288	751	0.20	0.44	39350	134504	3.07 (2.09 - 4.05)
12 – 13	2537	697	0.24	0.35	30647	95154	2.73 (1.73 - 3.73)
13 - 14	1840	670	0.31	0.26	22367	64506	2.44 (1.37 - 3.51)
14 – 15	1170	461	0.33	0.18	15285	42140	2.30 (1.23 - 3.38)
15 – 16	709	305	0.35	0.12	10103	26855	2.19 (1.10 - 3.27)
16 – 17	404	170	0.35	0.08	6553	16752	2.11 (1.07 - 3.15)
17 – 18	234	108	0.37	0.05	4207	10199	1.97 (0.93 - 3.01)
18 - 19	126	59	0.38	0.03	2625	5992	1.85 (0.86 - 2.84)
19 – 20	67	32	0.38	0.02	1626	3368	1.67 (0.76 - 2.59)
20 – 21	35	21	0.46	0.01	956	1742	1.41 (0.51 - 2.30)
21 - 22	14	11	0.55	0.01	486	786	1.17 (0.29 - 2.05)
22 -23	3	3	1.00	0.00	300	300	1.00 (0.04 - 2.04)

Table S8: Current life table of Beagles registered in SIAC.

Age interval in years (x. x + 1)	Number living at age x (lx)	Number dying in (x.x+1) (dx)	Conditional Probability of death (qx)	Proportion surviving to age x	Number of dog-years lived at age x L(x)	Total number of dog-years lived from year x T(x)	Life expectancy for dogs in the age interval (x. x + 1) e(x)
0 - 1	4064	7	0,00	1,00	99914	908769	9,09 (8,92 - 9,25)
1 – 2	4057	21	0,01	1,00	99570	808856	8,10 (7,84 - 8,37)
2 – 3	4036	71	0,02	0,99	98447	709285	7,14 (6,69 - 7,60)
3 - 4	3965	198	0,05	0,98	95204	610839	6,26 (5,55 - 6,97)
4 – 5	3767	360	0,09	0,93	88593	515635	5,55 (4,63 - 6,47)
5 – 6	3407	405	0,11	0,84	79627	427042	5,06 (4,09 - 6,03)
6 – 7	3002	409	0,13	0,75	70118	347415	4,64 (3,65 - 5,63)
7 – 8	2593	394	0,14	0,65	60728	277297	4,24 (3,26 - 5,23)
8 – 9	2199	356	0,15	0,56	51912	216569	3,86 (2,90 - 4,82)
9 - 10	1843	367	0,18	0,48	43390	164657	3,45 (2,46 - 4,44)
10 – 11	1476	388	0,23	0,39	34531	121268	3,10 (2,04 - 4,17)
11 – 12	1088	321	0,26	0,30	26137	86737	2,89 (1,81 - 3,97)
12 – 13	767	259	0,29	0,22	19063	60600	2,72 (1,60 - 3,84)
13 - 14	508	176	0,30	0,16	13505	41537	2,62 (1,52 - 3,72)
14 – 15	332	124	0,31	0,11	9408	28033	2,51 (1,40 - 3,62)
15 – 16	208	75	0,31	0,08	6483	18625	2,43 (1,37 - 3,50)
16 – 17	133	55	0,34	0,05	4403	12142	2,28 (1,19 - 3,38)
17 – 18	78	30	0,32	0,03	2930	7739	2,22 (1,19 - 3,24)
18 - 19	48	15	0,27	0,02	2046	4809	2,03 (1,18 - 2,88)
19 – 20	33	14	0,35	0,02	1424	2763	1,60 (0,79 - 2,41)
20 – 21	19	14	0,54	0,01	820	1338	1,19 (0,32 - 2,06)
21 - 22	5	5	1,00	0,01	518	518	1,00 (0,02 - 1,98)

Table S9: Current life table of German Shorthaired Pointers registered in SIAC.

Age interval in years (x. x + 1)	Number living at age x (lx)	Number dying in (x.x+1) (dx)	Conditional Probability of death (qx)	Proportion surviving to age x	Number of dog-years lived at age x L(x)	Total number of dog-years lived from year x T(x)	Life expectancy for dogs in the age interval (x. x + 1) e(x)
0 - 1	4852	68	0.01	1.00	99304	883454	8.83 (8.38 - 9.29)
1 – 2	4784	220	0.04	0.99	96392	784150	7.95 (7.17 - 8.74)
2 – 3	4564	309	0.07	0.94	91092	688688	7.31 (6.40 - 8.22)
3 - 4	4255	304	0.07	0.88	84973	598085	6.80 (5.90 - 7.69)
4 – 5	3951	293	0.07	0.82	79008	513365	6.27 (5.40 - 7.14)
5 – 6	3658	264	0.07	0.76	73429	434525	5.71 (4.90 - 6.52)
6 – 7	3394	322	0.09	0.71	67573	361097	5.10 (4.23 - 5.97)
7 – 8	3072	355	0.11	0.64	60852	293523	4.56 (3.66 - 5.46)
8 – 9	2717	371	0.13	0.57	53672	232671	4.06 (3.15 - 4.97)
9 - 10	2346	389	0.15	0.50	46179	179000	3.58 (2.66 - 4.50)
10 – 11	1957	403	0.19	0.42	38397	132821	3.14 (2.19 - 4.08)
11 – 12	1554	440	0.25	0.34	30172	94424	2.74 (1.72 - 3.76)
12 – 13	1114	399	0.30	0.26	21966	64253	2.48 (1.40 - 3.56)
13 - 14	715	273	0.32	0.18	15142	42286	2.34 (1.27 - 3.42)
14 – 15	442	194	0.36	0.12	10046	27144	2.22 (1.10 - 3.33)
15 – 16	248	104	0.35	0.08	6482	17098	2.18 (1.11 - 3.25)
16 – 17	144	56	0.33	0.05	4289	10615	2.07 (1.09 - 3.05)
17 – 18	88	41	0.38	0.03	2802	6326	1.83 (0.85 - 2.81)
18 - 19	47	26	0.43	0.02	1684	3524	1.64 (0.65 - 2.63)
19 – 20	21	13	0.47	0.01	930	1840	1.51 (0.52 - 2.50)
20 – 21	8	5	0.48	0.01	489	910	1.42 (0.48 - 2.35)
21 - 22	3	2	0.50	0.00	252	421	1.25 (0.40 - 2.10)
22 -23	1	1	1.00	0.00	168	168	1.00 (0.02 - 1.98)

Table S10: Current life table of Poodles registered in SIAC.

Age interval in years (x. x + 1)	Number living at age x (lx)	Number dying in (x.x+1) (dx)	Conditional Probability of death (qx)	Proportion surviving to age x	Number of dog-years lived at age x L(x)	Total number of dog-years lived from year x T(x)	Life expectancy for dogs in the age interval (x. x + 1) e(x)
0 - 1	4116	79	0.02	1.00	99049	1084827	10.85 (10.25 - 11.45)
1 – 2	4037	121	0.03	0.98	96650	985777	10.05 (9.33 - 10.77)
2 – 3	3916	135	0.03	0.95	93589	889127	9.34 (8.60 - 10.08)
3 - 4	3781	135	0.04	0.92	90362	795538	8.65 (7.92 - 9.37)
4 – 5	3646	152	0.04	0.89	86937	705176	7.95 (7.20 - 8.69)
5 – 6	3494	180	0.05	0.85	82987	618239	7.26 (6.47 - 8.05)
6 – 7	3314	191	0.06	0.81	78585	535251	6.62 (5.83 - 7.41)
7 – 8	3123	190	0.06	0.76	74067	456666	5.98 (5.22 - 6.75)
8 – 9	2933	201	0.07	0.72	69435	382599	5.33 (4.57 - 6.09)
9 - 10	2732	253	0.09	0.67	64088	313164	4.67 (3.86 - 5.48)
10 – 11	2479	319	0.12	0.61	57426	249075	4.08 (3.19 - 4.96)
11 – 12	2160	358	0.15	0.54	49619	191649	3.57 (2.65 - 4.49)
12 – 13	1802	358	0.18	0.46	41395	142030	3.12 (2.20 - 4.04)
13 - 14	1444	340	0.21	0.37	33356	100635	2.70 (1.79 - 3.61)
14 – 15	1104	343	0.27	0.29	25472	67278	2.29 (1.35 - 3.22)
15 – 16	761	330	0.36	0.22	17682	41806	1.94 (0.95 - 2.93)
16 – 17	431	225	0.41	0.14	10982	24124	1.74 (0.73 - 2.75)
17 – 18	206	129	0.48	0.08	6180	13143	1.62 (0.55 - 2.69)
18 - 19	77	47	0.47	0.04	3252	6963	1.64 (0.58 - 2.70)
19 – 20	30	16	0.42	0.02	1784	3710	1.64 (0.67 - 2.61)
20 – 21	14	8	0.44	0.01	1018	1926	1.47 (0.56 - 2.38)
21 - 22	6	4	0.50	0.01	545	909	1.25 (0.40 - 2.10)
22 -23	2	2	1.00	0.00	363	363	1.00 (0.02 - 1.98)

Table S11: Current life table of Estrela Mountain Dogs registered in SIAC.

Age interval in years (x. x + 1)	Number living at age x (lx)	Number dying in (x.x+1) (dx)	Conditional Probability of death (qx)	Proportion surviving to age x	Number of dog-years lived at age x L(x)	Total number of dog-years lived from year x T(x)	Life expectancy for dogs in the age interval (x. x + 1) e(x)
0 - 1	3536	197	0,05	1.00	100000	1,00	7,50 (6,66 - 8,33)
1 – 2	3339	247	0,07	0.95	94580	0,95	6,90 (5,98 - 7,82)
2 – 3	3091	254	0,08	0.88	87833	0,88	6,39 (5,46 - 7,32)
3 - 4	2837	242	0,08	0.81	80900	0,81	5,90 (5,00 - 6,80)
4 – 5	2595	244	0,09	0.74	74281	0,74	5,38 (4,48 - 6,27)
5 – 6	2350	235	0,10	0.68	67611	0,68	4,86 (3,99 - 5,73)
6 – 7	2115	261	0,12	0.61	61172	0,61	4,32 (3,42 - 5,21)
7 – 8	1854	216	0,11	0.54	54061	0,54	3,82 (3,02 - 4,62)
8 – 9	1638	255	0,14	0.48	48110	0,48	3,23 (2,40 - 4,06)
9 - 10	1383	299	0,20	0.41	41161	0,41	2,69 (1,82 - 3,56)
10 – 11	1084	317	0,26	0.33	33130	0,33	2,22 (1,34 - 3,10)
11 – 12	766	326	0,35	0.25	24678	0,25	1,81 (0,89 - 2,73)
12 – 13	441	251	0,44	0.16	16018	0,16	1,52 (0,58 - 2,46)
13 - 14	190	131	0,51	0.09	8921	0,09	1,33 (0,39 - 2,27)
14 – 15	59	46	0,56	0.04	4347	0,04	1,21 (0,28 - 2,14)
15 – 16	12	10	0,59	0.02	1908	0,02	1,12 (0,22 - 2,01)
16 – 17	2	2	1,00	0.01	786	0,01	1,00 (0,02 - 1,98)

Table S12: Current life table of Yorkshires Terrier registered in SIAC.

Age interval in years (x. x + 1)	Number living at age x (lx)	Number dying in (x.x+1) (dx)	Conditional Probability of death (qx)	Proportion surviving to age x	Number of dog-years lived at age x L(x)	Total number of dog-years lived from year x T(x)	Life expectancy for dogs in the age interval (x. x + 1) e(x)
0 - 1	3533	4	0.02	1.00	99942	1170142	11.70 (11.55 - 11.86)
1 – 2	3529	6	0.03	1.00	99798	1070200	10.71 (10.53 - 10.89)
2 – 3	3523	10	0.03	1.00	99576	970402	9.73 (9.52 - 9.95)
3 - 4	3513	21	0.04	0.99	99143	870826	8.76 (8.46 - 9.06)
4 – 5	3492	42	0.04	0.99	98260	771683	7.81 (7.41 - 8.20)
5 – 6	3451	105	0.05	0.98	96218	673423	6.89 (6.31 - 7.48)
6 – 7	3346	196	0.06	0.95	92064	577205	6.09 (5.33 - 6.85)
7 – 8	3150	223	0.06	0.89	86315	485141	5.43 (4.65 - 6.21)
8 – 9	2927	299	0.07	0.83	79219	398825	4.79 (3.92 - 5.66)
9 - 10	2628	299	0.09	0.75	71125	319606	4.25 (3.40 - 5.10)
10 – 11	2329	348	0.12	0.67	62412	248481	3.70 (2.81 - 4.60)
11 – 12	1981	361	0.15	0.58	52928	186069	3.22 (2.32 - 4.12)
12 – 13	1620	398	0.18	0.48	42843	133141	2.77 (1.82 - 3.72)
13 - 14	1222	349	0.21	0.38	32885	90298	2.40 (1.48 - 3.33)
14 – 15	873	340	0.27	0.28	23595	57413	2.04 (1.07 - 3.00)
15 – 16	533	278	0.36	0.19	15071	33818	1.78 (0.75 - 2.81)
16 – 17	255	150	0.41	0.11	8610	18747	1.68 (0.62 - 2.74)
17 – 18	105	69	0.48	0.06	4576	10137	1.67 (0.53 - 2.81)
18 - 19	36	20	0.47	0.03	2406	5561	1.81 (0.72 - 2.90)
19 – 20	16	6	0.42	0.02	1466	3155	1.81 (0.96 - 2.66)
20 – 21	10	6	0.44	0.01	919	1689	1.42 (0.52 - 2.32)
21 - 22	4	3	0.50	0.01	472	770	1.19 (0.32 - 2.06)
22 -23	1	1	1.00	0.00	298	298	1.00 (0.02 - 2.02)
